# Ductile and brittle transition behavior of titanium alloys in ultra-precision machining

**DOI:** 10.1038/s41598-018-22329-2

**Published:** 2018-03-02

**Authors:** W. S. Yip, S. To

**Affiliations:** State Key Laboratory in Ultra-precision Machining Technology, Department of Industrial and Systems Engineering, The Hong Kong Polytechnic University, Hung Hom, Kowloon, Hong Kong SAR, China

## Abstract

Titanium alloys are extensively applied in biomedical industries due to their excellent material properties. However, they are recognized as difficult to cut materials due to their low thermal conductivity, which induces a complexity to their deformation mechanisms and restricts precise productions. This paper presents a new observation about the removal regime of titanium alloys. The experimental results, including the chip formation, thrust force signal and surface profile, showed that there was a critical cutting distance to achieve better surface integrity of machined surface. The machined areas with better surface roughness were located before the clear transition point, defining as the ductile to brittle transition. The machined area at the brittle region displayed the fracture deformation which showed cracks on the surface edge. The relationship between depth of cut and the ductile to brittle transaction behavior of titanium alloys in ultra-precision machining(UPM) was also revealed in this study, it showed that the ductile to brittle transaction behavior of titanium alloys occurred mainly at relatively small depth of cut. The study firstly defines the ductile to brittle transition behavior of titanium alloys in UPM, contributing the information of ductile machining as an optimal machining condition for precise productions of titanium alloys.

## Introduction

Titanium alloys are applied widely in aerospace and medical industries because of their superior material properties such as high strength and fracture resistance^1–4^. They become one of the most popular medical alloys and they are used in different complex forms such as cylinder, bar, plate, sheet and stripe^5^ in the real applications. They are required to machine precisely for obtaining the desired shape and an excellent surface quality in order to fulfill the medical uses. Titanium alloys hold a high yield strength and their flow stresses increase suddenly when the strain rate is over 10^3^ s^−1^ in machining processes^6,7^, adding the challenges in the machining process. The deformation mechanism of titanium alloys in machining is extremely complicated and deviates from the other traditional alloys such as aluminum alloys and magnesium alloys^8^.

The low thermal conductivity of titanium alloy leads to an ineffective heat transference from the cutting zone to the surrounding^9–12^, causing an ineffective dissipation of cutting heat from the tool/workpiece interface. On the other hand, the ability of sustaining strength at elevated temperature of titanium alloys, the resulting working hardening on the machined surface is serious^8^ which further induces the high cutting force and the intensive cutting force variation^3^. High cutting temperature in machining processes is another problematic issue and places negative influences on machining performances^13,14^. Also, the phase change caused by the high cutting heat in machining leads the alternation of the deformation regime in machining processes, adding the difficulty on machining of titanium alloys especially in ultra-precision machining (UPM). In general, the machining mechanism of a material in a cutting process is mainly evaluated by the machining indicators of chip formation and tool wear^15–17^. Because of the low thermal conductivity and the extremely high strain rate induced in the machining processes of titanium alloys, the chip formation happens rapidly in the material removal process^18^. On the other hand, the high material shearing stress and plastic deformation with extremely high cutting temperature affect the chip formation^18^. Consequently, the chip formation in machining of titanium alloys is influenced by many machining factors and therefore it is difficult to conduct an analysis of cutting mechanism of titanium alloys. On the other hand, tool wear in machining of titanium alloys is serious and tool life is reported to be short^19,20^. The tool damage leads to the dramatic change in experimental conditions and the interrupt cutting motion in the cutting processes. Because of the frequent tool damage, the machining mechanism of titanium alloys is difficult to observe as the change in the tool condition causes the alteration of cutting mechanism. Moreover, the machined materials around the tool/workpiece interface are suffered from physical and metallurgical changes because of high cutting temperature^18^. In response to the above uncertainty in observing the machining indicators in machining of titanium alloys, the cutting mechanism of titanium alloys is always difficult to determine.

Cutting a groove with tilted angle was a common methodology for determining the deformation regime of brittle materials in previous literature^21–24^, however, it is not feasible for the case of identifying the deformation characteristic of titanium alloys which are hard and exhibit low thermal conductivity. For the low thermal conductivity materials, the thermal gradient and thermal softening effects at the tool/workpiece interface in machining processes are always dominant and accumulative; when titanium alloys are cut with an increasing depth of cut, the dynamic increase of depth of cut provides the extra fresh materials and surface areas in the machined surface, they develop an averaging effect on the cutting zone as the fresh surfaces serve as new cooling media. In this study, we cut an individual groove with different depth of cut instead of an increasing depth of cut at a single groove. In response to the material property of low thermal conductivity of titanium alloys, a sharp transition point is expected to appear on the machined surface under cutting at the same level of depth of cut.

The ductile to brittle behavior of brittle materials has been extensively investigated previously^25,26^, and the ductile machining strategy is widely adopted to improve surface finishing of the brittles materials. However, up to now, limited researches have reported the machining characteristics of titanium alloys, the fracture mechanism of these alloys in the material removal process in UPM is ever fewer reported. This paper aims to reveal the ductile to brittle transition regime of titanium alloys in UPM and shows the effects of depth of cut on the critical cutting distance and the ductile to brittle transition behavior of titanium alloys. We reported that there was a ductile deformation region on the machined surface of titanium alloys which showed a lower level of elastic recovery and better surface integrity, and it was highly depended on depth of cut. The cutting area outside the ductile deformation region suffered from a higher level of material swelling and the brittle fracture deformation, resulting of poorer surface integrity and the generation of uncut materials, which was defined as a brittle deformation region. The experimental results of cutting force, cutting profile, surface roughness, chip formation and machined surface in the different areas of machined surface were investigated and they gave out the evidences of above phenomenon. The clear crack was appeared at the ductile and brittle transition point which located in between of the machined surface of lower and higher elastic recovery. The cutting distance that started to appear the brittle fracture was denoted as the critical cutting distance. The present study firstly defines the ductile to brittle deformation mechanism and the critical cutting distance of titanium alloys in UPM, providing the information of optimal machining condition in precision machining of titanium alloys.

## Method

**Figure 1:**
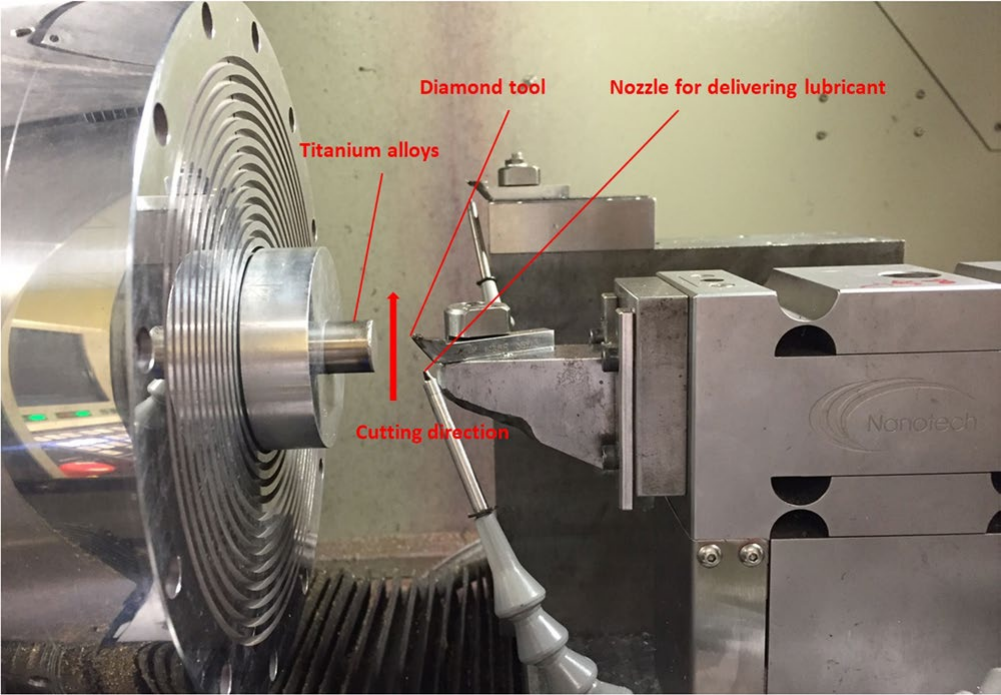
Experimental Setup of diamond cutting for generating a machined groove. The upward movement of diamond tool provided the cutting motion on the machined surface.

Titanium alloys Ti6Al4V (TC4) which contain 0.25% of iron, 0.2% of oxygen, 6% aluminum, 4% of beta phase stabilizer, 6% alpha phase stabilizer and remaining parts of titanium were used for the experiments. The length and diameter of titanium alloys were 40 mm and 16 mm respectively. Titanium alloys underwent straight line cuttings by the single point diamond tool. Depth of cut in the experiments was adjusted from 2 μm–7 μm with 1 μm interval value; therefore, six straight lines were cut individually on the titanium alloys’ surface with different depth of cut. Feedrate (cutting velocity) was set as 150 mm/min and unchanged throughout the experiments. The radius and height of diamond tool were 1.494 mm and 10.186 mm respectively. The chips were observed under scanning electron microscopy (SEM) machine Hitachi HT3030. The cutting force in three directions was captured by a force sensor Kistler 9256 C. Moore Nanotech 350FG (4 axis Ultra-precision machine) was used for diamond cutting. Surface roughness and the cutting profile of machined surface were measured by Wyko NT8000 Optical Profiling System, which is the optical profiler using non-contact measurement. The experimental setup is shown in Fig. 1. The fixture with the workpiece was hold on to the ultra-precision machine by the vacuum suction. The diamond tool moved in the upward direction and provided the straight line cutting on the workpiece, forming the groove on the machined surface.

## Results and Discussion

### Chip formation

**Figure 2:**
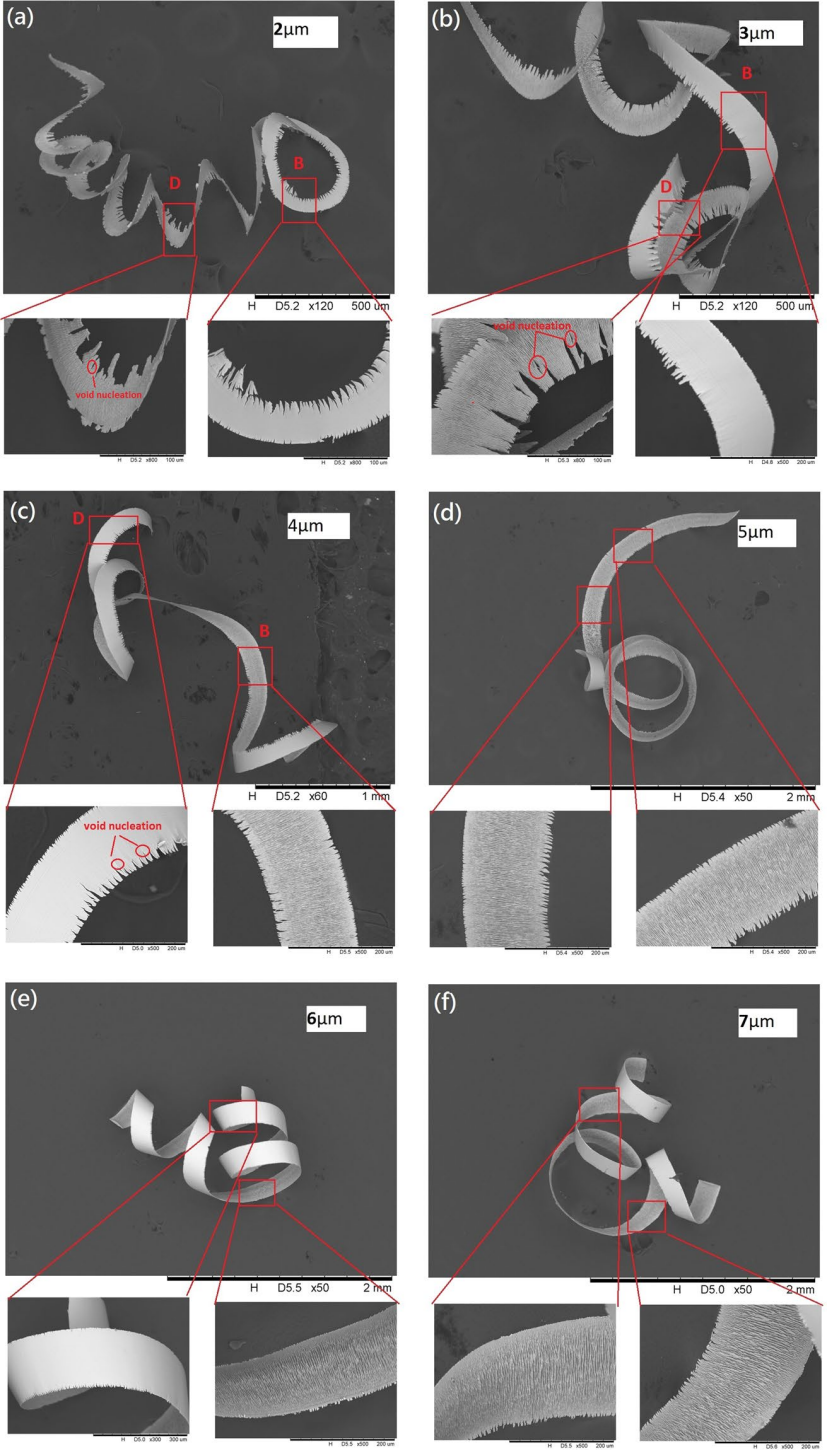
(**a**–**f**). The chip formations with the indication of brittle and ductile deformation areas at depth of cut 2 μm–7 μm. The letters B and D are the brittle deformation area and the ductile deformation area respectively.

The chip morphology and segmentation occupy the crucial roles in observing the machinability and the machining mechanism of titanium alloys. The chip formations generated at different depth of cut are shown in Fig. 2 (a–f). Cook^27^ explained the relationship between the chip morphology and cutting temperature in the cutting process of titanium alloys; when the thermal softening effect in the primary deformation zone was dominant over the strain hardening effect, the chip would appear as a serrated shape. According to Fig. 2(a), for the chip formation generated at depth of cut 2 μm, the length of saw tooth was the largest in comparison to that of generated at other depth of cut, this explained the thermal softening effect was stronger at relatively small depth of cut. The intensive thermal softening effect at the tool/workpiece interface further promoted the ductile deformation at the cutting zone, therefore, the length of saw-tooth decreased with depth of cut increase, and the degree of ductile deformation decreased with the cutting thickness increased.

The chip edge showed the saw-tooth shape at the cutting conditions of depth of cut 2 μm–4 μm, the saw-tooth edge gradually disappeared when depth of cut increased to 5 μm or above. Moreover, according to Fig. 2 (a–c), the void nucleation was observed near the chip edge at depth of cut 2 μm–4 μm; the void nucleation inside the chip suggests the ductile fracture in the cutting process^28–30^, which provided the evidence of the existence of ductile deformation at those depth of cut. For the chip formations generated at higher depth of cut 5 μm–7 μm as shown in Fig. 2 (d–f), the shapes of those chips were entire without any void, which implied that the brittle deformation was dominant over the whole cutting process without involving ductile mode cutting. The above experimental results explained that the cutting thickness governed the ductile and brittle deformation of UPM of titanium alloys.

On the other hand, the periodic saw-tooth was observed in the chip edge at depth of cut 2 μm–4 μm, which the length of saw-tooth in the chip edge was inhomogeneous in one single chip. The parts of chip edge displayed the longer saw-tooth while other parts displayed the shorter saw-tooth. This finding is matched with the report from Sun. S *et al*.^31^, which they observed that the periodic chip edge appeared in the cross section of chips in turning of titanium alloys. The periodic chip structures were disappeared when depth of cut increased to 5 μm–7 μm. The generations of inhomogeneous chip edge at depth of cut 2 μm–4 μm indicated that the brittle and ductile deformation occurred simultaneously at one single cut at relatively small depth of cut, similarly, the generations of homogeneous chip edge at depth of cut 5 μm–7 μm implied that the brittle deformation was the only deformation mechanism at relatively large depth of cut. The corresponding areas of brittle and ductile deformations on the chip edge are denoted as letters “B” and “D” respectively in Fig. 2 (a–f).

### Cutting profile and surface quality

**Figure 3:**
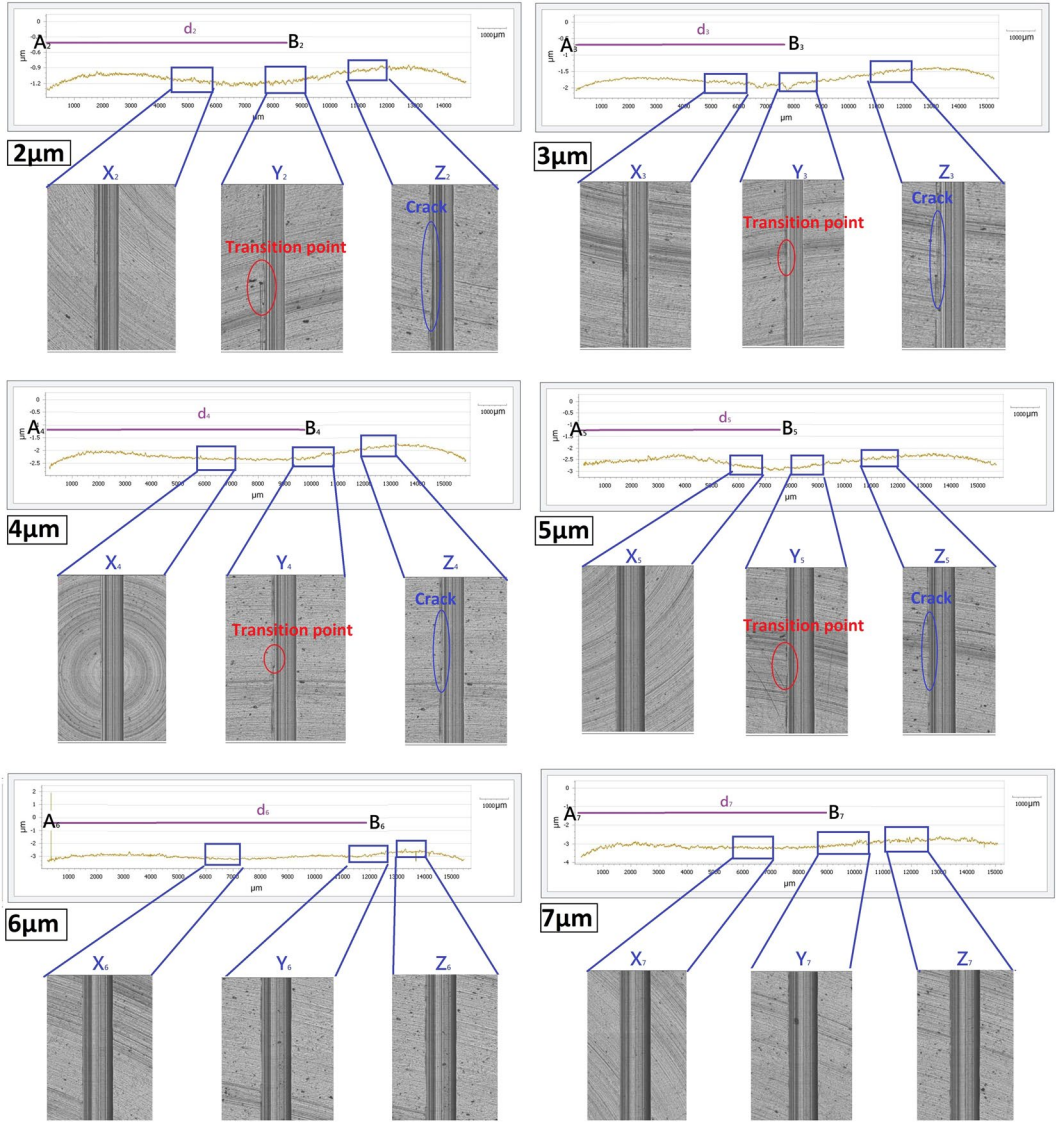
The groove profiles and groove surfaces generated at depth of cut 2 μm–7 μm. The letters X, Y and Z are denoted as the area with “a lower level of material swelling”, “a transition point of lower material swelling to higher material swelling” and “a higher level material swelling”.

The cutting profiles of machined groove at depth of cut 2 μm–7 μm are shown in Fig. 3. According to Fig. 3, the cutting profiles of every individual machined groove (2 μm–7 μm) showed various depth along a constant depth of cut, which was caused by the material swelling effect. Actually, the material swelling effect commonly occurs in UPM. The materials that are melted become viscous fluid under continuous cutting and the material fluid localizes at the two sides and the bottom of the tool edge. The metal fluid solidifies and expands when temperature of the machined surface decreases^32,33^, as a result, the machined surface swells and apparent tool marks are left on the machined surface, introducing the deviation of actual cutting depth from the assigned one. The microscopes of three areas of each machined groove were captured, they are denoted as X, Y and Z as the figures shown. The letters of X, Y and Z with the subscript of depth of cut denoted as the machined areas with “a lower level of material swelling”, “a transition point of lower material swelling to higher material swelling” and “a higher level material swelling” respectively. The depth variation among the individual machined groove was different from that of each other, and it is highly depended on depth of cut. According to Fig. 3, for depth of cut 2 μm–4 μm, the depth of machined groove varied along the machined surface, the depth of machined groove firstly decreased and then reached to the lowest value, the depth increased again in the following cutting distance, demonstrating the different degrees of material swelling at one single cut. For the machined groove generated at depth of cut 5 μm–7 μm, the cutting profiles of them appeared distinctively, the decreasing/increasing rate of depth in the cutting profile was smaller. The above results suggested that the material swelling rate changed when depth of cut was various, and therefore, the cutting mechanism altered when depth of cut changed.

Areas X of all machined grooves displayed differently from areas Y and Z; areas X had the smoother and finer surfaces in comparison to that of the areas Y and X generated in all depth of cut, there was no tear on the surface and surface edge at areas X. For areas Y, there was a clear crack on the groove edge (shown as red circles in Fig. 3), it became the important indicator for the transition point from the lower material swelling area to the higher material swelling area and the start of ductile to brittle transition, the crack was defined as the transition point of ductile to brittle area. The machined surfaces of areas Z displayed coarseness, the left of groove edge at areas Z contained cracks (shown as blue circle in Fig. 3) and they were the footprints of uncut materials which were generated by the brittle deformation under the brittle fracture mode.

**Figure 4:**
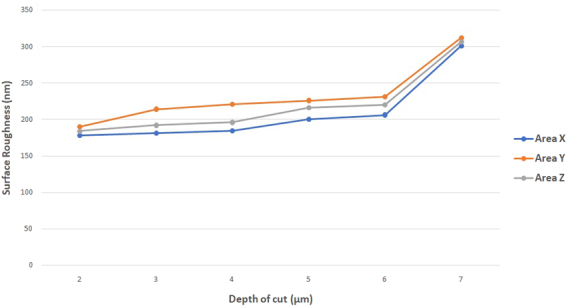
Surface roughness of areas X, Y and Z generated at depth of cut 2 μm–7 μm.

Surface roughness of areas X, Y and Z at depth of cut 2 μm–7 μm is shown in Fig. 4. The results are consistent with the cutting profiles shown in Fig. 3, because of the brittle deformation and fracture tearing of chip at areas Z, it could been seen that surface finishing obtained in areas X was superior than that of areas Z, which surface roughness of areas X was always smaller than that of areas Z. Especially for the surface generated at depth of cut 3 μm and 4 μm, the difference of surface roughness between these two areas was remarkably large, this large variation was again counted by the different deformation modes at the area X (ductile) and the area Z (brittle). For areas Y at all depth of cut, surface roughness was the highest among other areas; the underlying reason was the formation of the crack at the ductile to brittle transition point, consequently, the ragged surface caused the worst surface quality.

**Figure 5:**
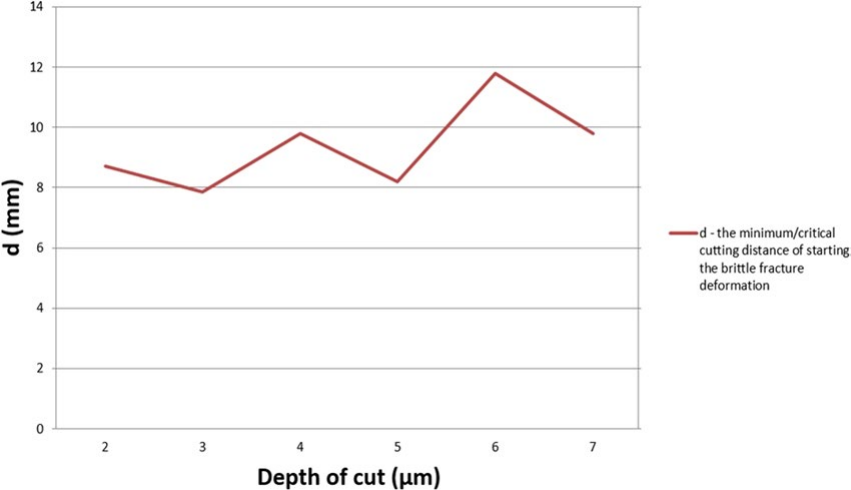
The graph of d versus different depth of cut. d is the minimum/critical cutting distance of starting the brittle fracture deformation.

The cutting distance between areas X and Y is expressed as d, it explained the minimum/critical cutting distance of the brittle fracture deformation and the start of coarsened surface appearance. The graph of d versus depth of cut is shown in Fig. 5. The minimum/critical cutting distance increased with depth of cut increase.

**Figure 6:**
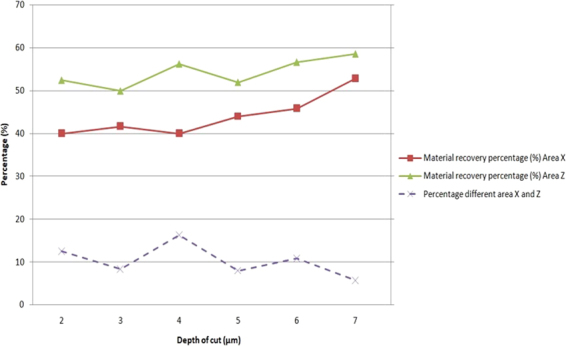
The material recovery percentage of areas X and Z.

Figure 6 shows the deep material swelling percentage of areas X and Z after the diamond cutting, which was determined by the difference between the assigned and actual cutting depth at the bottom of machined surface. Deep material swelling is caused by the expanded materials after the solidification of melted metal fluid in cutting. According to Fig. 6 the material swelling effect was serious for titanium alloys in UPM, the material swelling percentage was over at least 40% for both areas X and Z no matter the value of depth of cut, it is one of the important machining characteristics of titanium alloys, which is low elastic modulus materials.

### Cutting Force analysis

**Figure 7:**
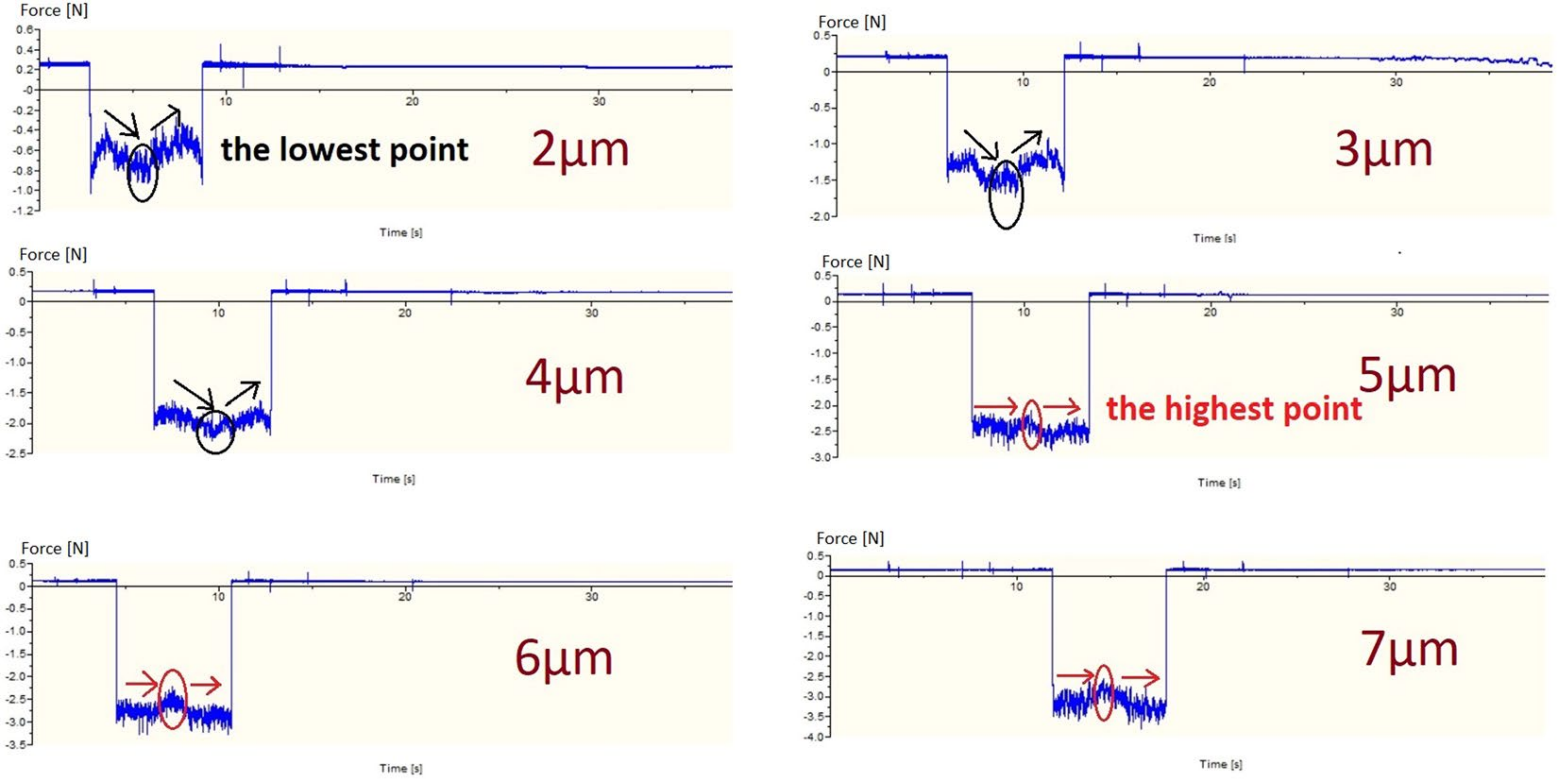
The thrust forces of machined grooves generated at depth of cut 2 μm–7 μm.

Figure 7 shows the thrust force of machined groove generated at depth of cut 2 μm–7 μm. The thrust force was used to identify the level of deep material swelling at the bottom area of the machined surface^9–11^. The higher value of thrust force means the higher level of deep material swelling on the machined surface and vice via. According to Fig. 7, the thrust force was varied through a single cut, it implied that the material swelling level was varied alone one machine groove and it was consistence to the different values of cutting depth on the individual machined groove as shown in Fig. 3. The variation of thrust force decreased with depth of cut increase. The thrust forces fluctuated largely at depth of cut 2 μm–4 μm, and it became flatter and more stable at depth of cut 5 μm–7 μm. In general, the implications of thrust force patterns in Fig. 7 were matched to the cutting profiles shown in Fig. 3.

As illustrating in Fig. 7, the patterns of increasing/decreasing trend of thrust force at depth of cut 2 μm–4 μm were different from that of depth of cut 5 μm–7 μm. For the thrust forces generated at depth of cut 2 μm–4 μm, the thrust forces firstly decreased and following with an increasing trend, the sharp lowest points were observed at the transition points. On the contrary, for the thrust forces at depth of cut 5 μm–7 μm, the thrust force patterns displayed dissimilarly, they showed relatively flat comparing to that of smaller depth of cut 2 μm–4 μm, which showed a comparable lower increasing/decreasing rate, and the highest sharp transition points were demonstrated in the thrust force signals. Actually, the increase and decrease of thrust forces at constant depth of cut implied that there were two cutting mechanisms at one single cut. The demonstration of relatively flat patterns in the thrust forces at depth of cut 5 μm–7 μm explained only one cutting mechanism throughout the whole cutting processes; with the information of chip formation and cutting profile discussed above, it can be concluded that the whole cutting processes conducted at depth of cut 5 μm–7 μm are described as brittle machining without a ductile material removal process.

## Conclusion

In this study, the cutting mechanism of titanium alloys in UPM was firstly revealed through experimental approaches. The experimental results displayed the existence of brittle and ductile deformation mechanism and the ductile to brittle transition behavior. The critical machining parameters for conducting ductile machining of titanium alloys are both the cutting distance and depth of cut. Ductile machining of titanium alloys in UPM occurs when the cutting distance is not over the critical cutting distance. The critical cutting distance increases with depth of cut increase. The ductile to brittle transition behavior mainly happens at smaller depth of cut (in this study, the depth of cut was 2 μm–4 μm), cutting at higher depth of cut would result of the single brittle removal mode. This study provides the important information of implementing ductile machining of titanium alloys in UPM, supporting to improve surface finishing and surface integrity of precise medical components.

### Data availability statement

The datasets generated during and/or analysed during the current study are available from the corresponding author on reasonable request.
